# Erotic Hallucinations Due to Cocaine Pressure Anesthesia

**Published:** 1913-08-15

**Authors:** 


### EROTIC HALLUCINATIONS DUE TO COCAINE PRESSURE ANESTHESIA.

The following from the *Dominion Dental Journal* by way of the *Dental Brief*, should be read and considered by all of those, without exception, who have to do with anesthetics, even the simple administration of cocaine as cited:

"Professor V. E. Henderson, of the University of Toronto, Canada, in the March number of the *Dominion Dental Journal*, relates a case that should serve as a warning to all those who use cocaine as a local anesthetic, to be careful to safeguard themselves by having a third party present, as they do when administering a general anesthetic.

"A dentist treating a small, somewhat nervous girl of sixteen years, found a molar tooth with an exposed pulp, and proceeded, after cleansing the cavity without causing pain, to apply a paste of cocaine crystals and alcohol, covering it with unvulcanized rubber and applying pressure in the usual manner, and in a few minutes removed the pulp painlessly. An appropriate dressing and temporary filling were then inserted and the patient dismissed.

"The next day she returned with her mother and brother-in-law and charged him with placing his hands up her clothes, touching her skin, and with other improprieties. The complainants demanded an apology, and one not being forthcoming, the matter was carried to the police and a charge made of indecent assault. A trial resulted. The evidence sifted down to the charge made by complainant and the denial of the defendant, and the judge in dismissing the prisoner, stated that he would not consider the expert testimony offered, but that as it was the unsubstantiated story of the man against that of the girl, he had no course open to him but to dismiss the prisoner.

"Professor Henderson states that this is the first case of the kind to his knowledge described in dental or medical literature in the English language, and points out the importance of this defect being rectified by any members of these professions who happen to know of pertinent cases. He gives numerous references to cases of erotic hallucination following the use of cocaine as a local anesthetic reported in foreign journals, sufficient to establish the fact of this possible effect of local cocaine anesthesia, and suggests that it should be more generally known and guarded against. This publication is timely as various methods of using anesthetic agents to make dental operations painless are coming to the fore. The fact that consciousness is not lost has usually been considered a factor of safety. Cocaine hallucinations do not immediately follow its administration, but may come on some time later, after the patient has returned home. Timely precautions may save much trouble; they are in order whenever anesthetic agents are used, as the old adage reads, 'An ounce of prevention, etc.'—W. H. T."

